# RDBridge: a knowledge graph of rare diseases based on large-scale text mining

**DOI:** 10.1093/bioinformatics/btad440

**Published:** 2023-07-17

**Authors:** Huadong Xing, Dachuan Zhang, Pengli Cai, Rui Zhang, Qian-Nan Hu

**Affiliations:** CAS Key Laboratory of Computational Biology, Shanghai Institute of Nutrition and Health, University of Chinese Academy of Sciences, Chinese Academy of Sciences, Shanghai 200031, China; Institute of Environmental Engineering, ETH Zurich, Zurich 8093, Switzerland; CAS Key Laboratory of Computational Biology, Shanghai Institute of Nutrition and Health, University of Chinese Academy of Sciences, Chinese Academy of Sciences, Shanghai 200031, China; CAS Key Laboratory of Computational Biology, Shanghai Institute of Nutrition and Health, University of Chinese Academy of Sciences, Chinese Academy of Sciences, Shanghai 200031, China; CAS Key Laboratory of Computational Biology, Shanghai Institute of Nutrition and Health, University of Chinese Academy of Sciences, Chinese Academy of Sciences, Shanghai 200031, China

## Abstract

**Motivation:**

Despite low prevalence, rare diseases affect 300 million people worldwide. Research on pathogenesis and drug development lags due to limited commercial potential, insufficient epidemiological data, and a dearth of publications. The unique characteristics of rare diseases, including limited annotated data, intricate processes for extracting pertinent entity relationships, and difficulties in standardizing data, represent challenges for text mining.

**Results:**

We developed a rare disease data acquisition framework using text mining and knowledge graphs and constructed the most comprehensive rare disease knowledge graph to date, Rare Disease Bridge (RDBridge). RDBridge offers search functions for genes, potential drugs, pathways, literature, and medical imaging data that will support mechanistic research, drug development, diagnosis, and treatment for rare diseases.

**Availability and implementation:**

RDBridge is freely available at http://rdb.lifesynther.com/.

## 1 Introduction

Rare diseases are medical conditions with a very low prevalence, typically defined as affecting fewer than 200 000 individuals in the USA or 1 in 2000 individuals in Europe (Haendel *et al.* 2020). The pharmaceutical industry and the medical community often neglect these diseases due to the challenges associated with developing treatments for small patient populations. However, rare diseases encompass over 7000 different conditions that collectively affect ∼300 million people worldwide (Marwaha *et al.* 2022).

Although a few manually curated databases have been established (Putkowski 2010, Weinreich *et al.* 2008), and efforts have been made to clean and integrate these (Gupta *et al.* 2016, Hoskins 2022, Kuo *et al.* 2022), a scarcity of knowledge remains (Nguengang Wakap *et al.* 2020) due to the low efficiency and timeliness of manual annotation. Gathering and exploring multiple data sources remains a time-consuming task that requires significant expertise, and a considerable amount of potential knowledge remains trapped within unstructured text (Nadif and Role 2021).

As biological data and information continue to proliferate, the wealth of knowledge in the scientific literature is rapidly expanding. So far, relevant studies in PubMed have reached >34 million (Sayers *et al.* 2022). Manually extracting target information from such a massive document collection is unrealistic. However, with achievements in computer science, especially deep learning, the fusion of life science and computer technology makes it possible to mine information from millions of unstructured biomedical texts.

Text mining is an automated method for obtaining information that transforms unstructured text into a format that can reveal useful patterns and insights. It uses sophisticated methods, including natural language processing (NLP), machine learning, and statistics, to find and analyze hidden connections in textual data. Text mining has various applications, including sorting text into categories, grouping text by similarity, detecting emotions in text, creating summaries of text, and identifying relations between entities in text, enabling researchers to handle large-scale literature and unstructured text containing entities of interest.

While scientific text mining has achieved significant progress, applications in biomedicine, particularly in the field of rare diseases, remain limited (Davenport and Kalakota 2019, Renganathan 2017). Several challenges must be addressed. Firstly, training deep learning models for text mining requires annotated data, but rare diseases typically have insufficient annotated data, making it challenging to train accurate models. Secondly, biological mechanisms and pathways of rare diseases are often poorly understood, hindering extraction of information and creation of accurate models for text mining. Thirdly, standardizing data extracted from the literature on rare diseases is challenging due to a lack of references and standards. Finally, text mining data are often stored in databases where the information can be challenging to interpret intuitively, particularly for complex data, such as pathways and images.

**Figure 1.**
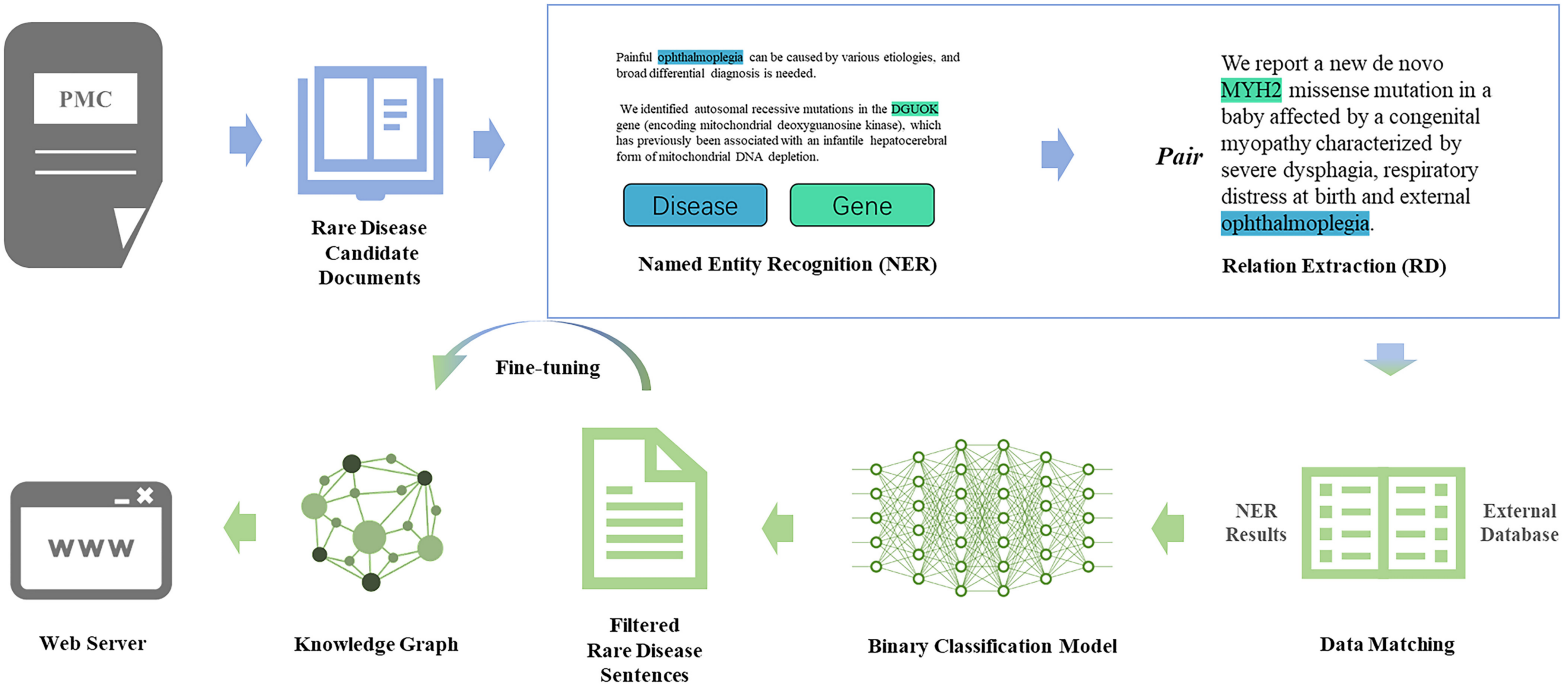
Framework of RDBridge. The workflow comprises a comprehensive series of stages designed to systematically process scientific documents to extract rare disease information. These stages encompass several critical processes, including document downloading, pre-processing, named entity recognition, relation extraction, data matching, binary classification model training, model retraining, knowledge discovery, and the use of web servers.

In this work, we developed a text mining framework for rare diseases to address these difficulties and tackle the complexity of this task (Fig. 1). We designed text-mining strategies for use with rare diseases and the associated genes, potential drugs, metabolic pathways, and medical images, integrating these into a comprehensive rare disease knowledge graph available as a web service, RDBridge. We believe that our approach will contribute significantly to the field of rare disease research and help accelerate the discovery of effective treatments for patients.

## 2 Methods

The full PMC Open Access Subset was utilized for text mining and downloaded in XML and Image format from the NCBI FTP site (https://www.ncbi.nlm.nih.gov/pmc/tools/ftp/). We selected keywords (Supplementary Keywords S1) and used regular matching algorithms to obtain 50 000 documents with the largest number of keywords as pre-processing data for entity and relationship extraction.

BioALBERT (Naseem *et al.* 2022) is a pre-trained deep learning model that can be used for various NLP tasks, including Named Entity Recognition (NER) and Relation Extraction (RE). For NER of diseases, genes, and potential drugs, the model was fine-tuned on specific datasets (Supplementary Table S1) to extract the entities from the pre-processing data. We matched extracted disease entities with rare diseases in the OMIM database (https://www.omim.org/). An equal number of entities that did not match diseases in OMIM were extracted from non-candidate documents. Paragraphs with matching entities were labeled as positive examples and paragraphs with non-matching entities as negative examples. The maximum token length of a paragraph was 512. A binary classification ResNet-50 (He *et al.* 2016) model was trained to determine whether a certain paragraph was related to a rare disease. The binary classification model was used to filter paragraphs with NER results from the pre-processing data, and then positive paragraphs and NER results were selected to fine-tune the BioALBERT model, resulting in a model suitable for rare disease NER, which was applied to extract entities from PMC data.

For RE of genes and potential drugs, the BioALBERT model was fine-tuned to extract relations between entities with datasets, then fine-tuned again based on the binary classification model and filtered corpus to obtain an RE model for rare diseases. When judging whether a sentence was related to a rare disease, we used the same model and parameters as those for NER. The ConsensusPath database (Kipf and Welling 2016) was used to annotate documents through regular matching, resulting in a corpus of 300 pieces. We fine-tuned a BioALBERT1.0 (PubMed+PMC)-based pathway NER model using this dataset. By leveraging sentence-level co-occurrence relationships, we established disease-pathway pair links when the disease and pathway NER models generated results from the same sentence. To enhance visualization of pathway information, we associated pathways using WikiPathways (Martens *et al.* 2021).

For medical images, we fine-tuned the EfficientNet-B5 (Tan and Le 2019) model using the ROCO (Pelka *et al.* 2018) dataset to derive a binary classification model that can determine whether an image in a document related to rare diseases in NER or RE is a medical image.

We matched disease names with diseases in OMIM and genes, compounds, and pathways from resources (Supplementary Fig. S1).

The RDBridge web interface was implemented using Python in Ubuntu (18.04.2). We implemented a front-end web interface using Vue (https://vuejs.org/) and Bootstrap Web (https://getbootstrap.com/).

## 3 Results

We developed a novel framework that automates data collection and representation of rare diseases using text mining and knowledge graphs, extracting information from the literature, and applying targeted text mining to different rare disease entities. We used multistep fine-tuning and proposed classification models to address problems of dataset scarcity and difficult relationship extraction, while using knowledge graphs and web services to address data search and visualization.

We built a series of models for the target entities. Supplementary Table S2 shows the performance of all models in the current study that achieved top-tier performance. Using these models, we identified 11 704 rare diseases (4145 matched to OMIM), 3153 disease-gene relationships, 15 349 gene-compound relationships, 3791 disease-pathway relationships, 235 631 publications, and 90 249 medical images.

To assess data availability and reliability, we attempted to match all text-mining entities to existing databases (Supplementary Fig. S1), enabling us to obtain reliable and informative text-mining results on rare diseases.

We constructed a knowledge graph that captured the relationships between rare diseases and entities, including genes, compounds, pathways, and medical images. Our visualization platform, RDBridge, enables researchers to explore rare disease knowledge graphs. RDBridge can automatically match 50 candidate diseases that best match the input provided, giving information on genes, pathways, and medical images, as well as references and associated databases.

Compared to existing databases, such as Orphanet (Weinreich *et al.* 2008), NORD (Putkowski 2010), RareDDB (Gupta *et al.* 2016), RSDB (Kuo *et al.* 2022), GARD (Nguengang Wakap *et al.* 2020), and eRAM (Jia *et al.* 2018), RDBridge encompasses all data categories found in other databases, while also providing a web server (Supplementary Table S3). Moreover, RDBridge integrates medical image information and establishes connections with diseases. Regarding algorithms, RDBridge has replaced the manual collection method and developed a rare disease mining framework based on text mining technology. eRAM has also employed text mining techniques to mine rare disease-related entities. However, eRAM relies on a traditional pattern-based method instead of utilizing deep learning, and it indiscriminately scans all texts rather than focusing specifically on rare diseases.

We used amyotrophic lateral sclerosis (ALS) as an RDBridge case study (Supplementary Fig. S2). ALS is a rare disease with no cure (Feldman *et al.* 2022) and an average survival time after diagnosis of 2–4 years (Hobson and McDermott 2016). By inputting “Amyotrophic lateral sclerosis” in the “Genes & Compounds” section, ALS-related gene, potential drug, pathway, literature, and medical image data can be obtained, with results superior to those of Orphanet (Supplementary Fig. S3). This shows that RDBridge provides more comprehensive information on related genes, potential drugs (Supplementary Fig. S2A), related pathways (Supplementary Fig. S2B), related literature (Supplementary Fig. S2C), and medical images (Supplementary Fig. S2D).

## 4 Conclusion

Rare diseases have received more attention in recent years (Halley *et al.* 2022), with various initiatives and organizations working to promote awareness, research, and policy (Luque *et al.* 2022, Putkowski 2010). In this work, we developed a novel framework and constructed the most comprehensive knowledge graph of rare diseases to date, capable of supporting mechanistic research, drug development, and clinical diagnosis. We also developed a web server that enables access to and visualization of the rare disease knowledge graph. RDBridge provides a user-friendly interface for retrieving and visualizing various entities in the rare disease knowledge graph extracted through the rare disease information extraction framework. Users can select different target entities and enter the name of a disease to obtain search results and links to external databases. We anticipate that this framework will create new opportunities for acquiring rare disease data.

While RDBridge can provide potential pathways and drug candidates relating to rare diseases, experimental verification and clinical trials are still required, and as with text-mining tools generally, RDBridge cannot replace manual data collection. However, the framework of RDBridge has methodological significance, and the approach used can address difficulties in obtaining rare disease data. Further optimization may involve adding more data entities, using an improved deep learning algorithm with higher precision for more accurate relationship extraction and NER, and sorting the correlation between the entities obtained and rare diseases.

RDBridge is designed to continuously scan for new research papers and other data sources, implementing deep learning algorithms to extract information. Additionally, we plan to regularly review and update RDBridge every two years, ensuring it remains an up-to-date and comprehensive resource.

To achieve the above goals, we will increase the range of data sources. Given the rapid growth in the scientific literature, we plan to conduct text mining on more abstracts and full texts and combine other data mining methods, visualization methods, drug evaluation methods, and more accurate deep learning algorithms for further development of this approach.

## Supplementary Material

btad440_Supplementary_Data
