# Supplementary material for: RDBridge: a knowledge graph of rare diseases based on large-scale text mining
Source: Bioinformatics. 2023 Jul 17;39(7):btad440. doi: 10.1093/bioinformatics/btad440 (PMC10368801; doi:10.1093/bioinformatics/btad440)
Supplement: btad440_Supplementary_Data [file btad440_supplementary_data.docx]

**Supplementary Material**

**RDBridge: a knowledge graph of rare diseases based on large-scale text mining**

Huadong Xing^1^, Dachuan Zhang^2^, Pengli Cai^1^, Rui Zhang^1^, Qian-Nan Hu^1,*^

^1^ CAS Key Laboratory of Computational Biology, Shanghai Institute of Nutrition and Health, University of Chinese Academy of Sciences, Chinese Academy of Sciences, Shanghai 200031, China

^2^ Institute of Environmental Engineering, ETH Zurich, Zurich 8093, Switzerland

Corresponding author. Qian-Nan Hu. Email: qnhu@sibs.ac.cn; Tel.: +86-21-54920615;

ORCID: 0000-0001-5213-472X

**Keywords 1:** ‘Rare disease | Congenital disorders | Rare anomalies | Rare disorder syndrome | Rare disorder epidemiology | Genetic predisposition to disease | Orphan syndrome | Rare conditions | Rare disorder | Chromosomal abnormalities | Rare pediatric diseases | Undiagnosed diseases | Rare genetic disease | Low-prevalence disease | Ultra-rare diseases | Undiagnosed disease | Rare genetic syndromes | Genetic disease | Genetic disorders | Orphan disease | Rare disease treatment | Genetic disorder | Mendelian disease | Uncommon disease | Neurogenetic disorders | Genetic disorders of metabolism | Rare diseases | Rare syndrome | Rare condition | Rare disease registry | Mendelian disorders | Uncommon diseases | Rare disorders of metabolism | Rare syndromes | Rare disease registries | Mendelian disorder | Genetic diseases of the immune system | Inherited genetic disorders | Rare genetic diseases | Rare illnesses | Inherited diseases | Rare disorders | Rare diseases and disorders | Rare disease diagnosis | Genetic testing for hereditary diseases | Rare illness | Orphan diseases | Rare genetic disorders | Genetic counseling for rare diseases | Rare medical conditions’

**Table S1. Finetuning datasets employed**

We selected different datasets for fine-tuning of tasks and entities. The *Dataset* column indicates the dataset and source, *Task* gives the type of text mining task, *Entity* corresponds to the entity examined, and *Number of annotations (relations)* shows the quantity of data in each dataset.

| **Dataset** | **Task** | **Entity** | **Number of annotations (relations)** |
| --- | --- | --- | --- |
| BC5CDR (disease) (Li, et al., 2016) | NER | Disease | 12864 |
| NCBI (disease) (Gerner, et al., 2010) | NER | Disease | 6892 |
| BC5CDR (chemical) (Li, et al., 2016) | NER | Chemical | 15933 |
| CHEMDNER (Krallinger, et al., 2015) | NER | Chemical | 84355 |
| BC2GM (Ando, 2007) | NER | Gene | 24583 |
| GAD (Bravo, et al., 2015) | RE | Gene-disease | 5330 |
| EU-ADR (Van Mulligen, et al., 2012) | RE | Gene-disease | 355 |
| ChemDisGene (Zhang, et al., 2022) | RE | Chemical-Gene | 3911 |

**Table S2** **Performance of all models**

The *Entity* column indicates the entity extracted, *Task* gives the type of text mining task, and *F1 Score* shows the performance of all models.

| **Entity** | **Task** | **F1 score** |
| --- | --- | --- |
| Rare disease | NER | 84.51 |
| Gene | NER | 85.35 |
| Chem/Drug | NER | 91.66 |
| Pathway | NER | 89.91 |
| Rare disease-Gene | RE | 61.57 |
| Rare disease-Pathway | RE | 64.25 |
| Rare disease related sentence | binary classification | 96.27 |
| Medical Image | binary classification | 94.43 |

**Figure S1.** Entities and their relationships with associated databases. We conducted literature-based data extraction for five entities related to rare diseases: genes, compounds, pathways, medical images, and actual diseases. We established relationships between these entities, including rare disease-gene, gene-compound, disease-pathway, and disease-medical image relationships. By screening genes, we were able to identify compounds associated with rare diseases that represent potential drug candidates. We identified disease names from the corresponding entries in the OMIM database. We matched relevant genes, compounds, and pathways from various sources of biomedical information, including CHEBI(Hastings, et al., 2016), PubChem (Kim, et al., 2021), HGNC (Tweedie, et al., 2021), Ensembl (Howe, et al., 2021), Drugbank (Wishart, et al., 2018) , InChI (Goodman, et al., 2021) and WikiPathways(Martens, et al., 2021).


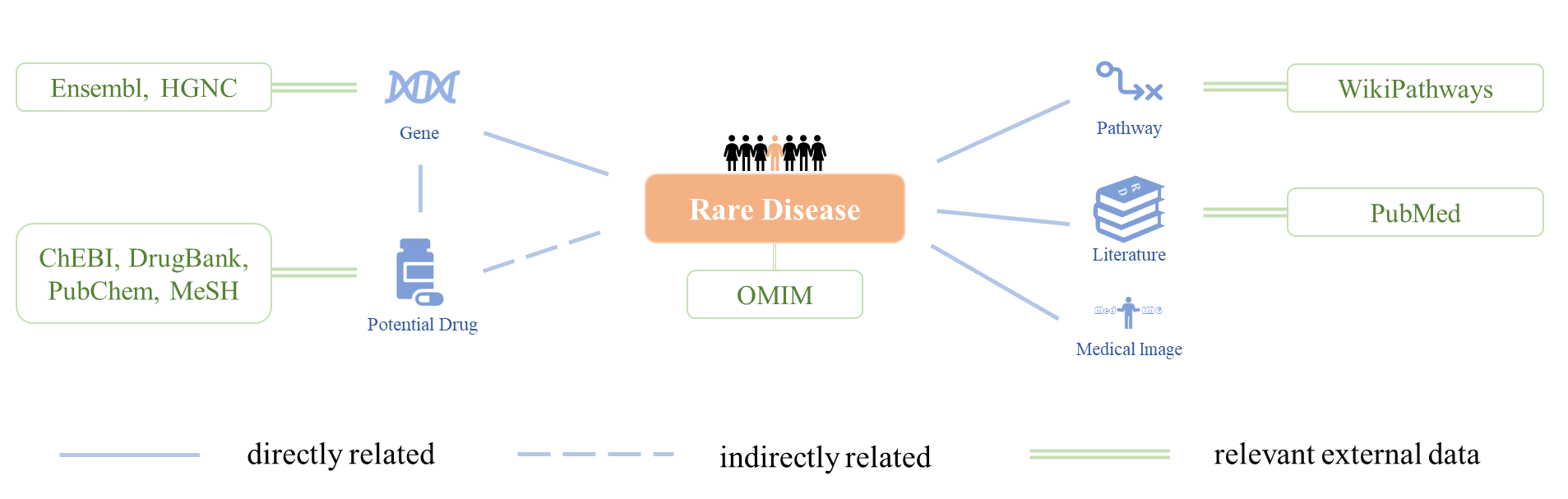


**Table S3 Database comparison for rare diseases**

We compared searchable entities in several existing rare disease databases. The *Database* column gives the name and source, *Type* indicates how data is obtained, and *Function* shows the searchable rare disease-related entities.

| **Database** | **Type** | **Function** | | | | |
| --- | --- | --- | --- | --- | --- | --- |
|  |  | *Gene* | *Compound/Drug* | *Pathway* | *Literature* | *Medical Image* |
| Orphanet (Weinreich, et al., 2008) | Manual collection | ✓ | ✓ | 🗶 | ✓ | 🗶 |
| NORD (Putkowski, 2010) | Manual collection | 🗶 | ✓ | 🗶 | ✓ | 🗶 |
| RareDDB (Gupta, et al., 2016) | Integrated database | ✓ | ✓ | ✓ | 🗶 | 🗶 |
| RSDB (Kuo, et al., 2022) | Integrated database | ✓ | ✓ | 🗶 | ✓ | 🗶 |
| GARD (Hoskins, 2022) | Manual collection | ✓ | ✓ | 🗶 | ✓ | 🗶 |
| eRAM (Jia, et al., 2018) | Text mining | ✓ | 🗶 | 🗶 | ✓ | 🗶 |
| RDBridge | Text mining | ✓ | ✓ | ✓ | ✓ | ✓ |

**Figure S2.** RDBridge web interface and examples for amyotrophic lateral sclerosis from RDBridge. (A) Genes and potential drugs. (B) Pathways. (C) Literature. (D) Medical images.


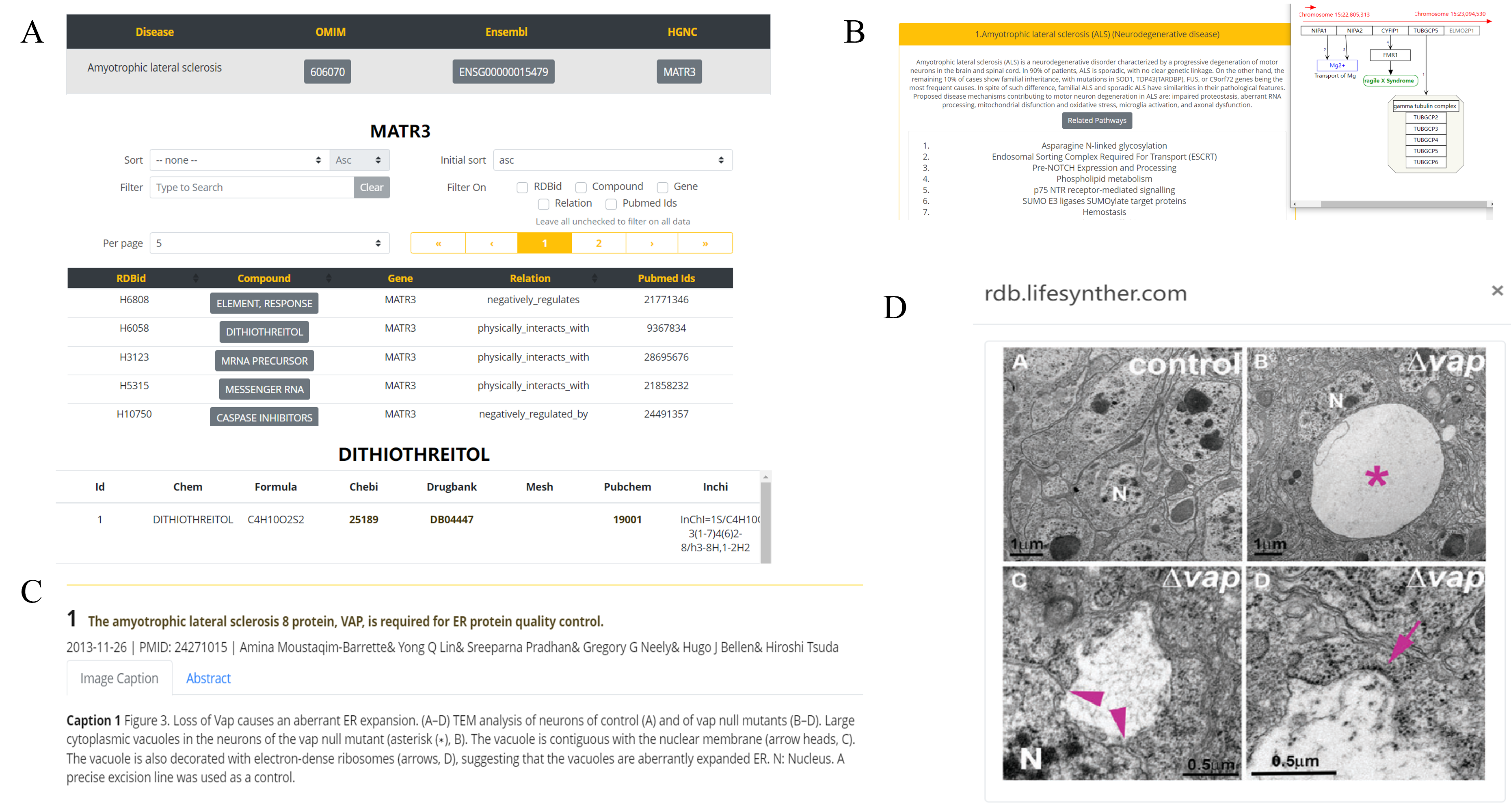


**Figure S3.** Amyotrophic lateral sclerosis (ALS) example from Orphanet (Weinreich, et al., 2008)


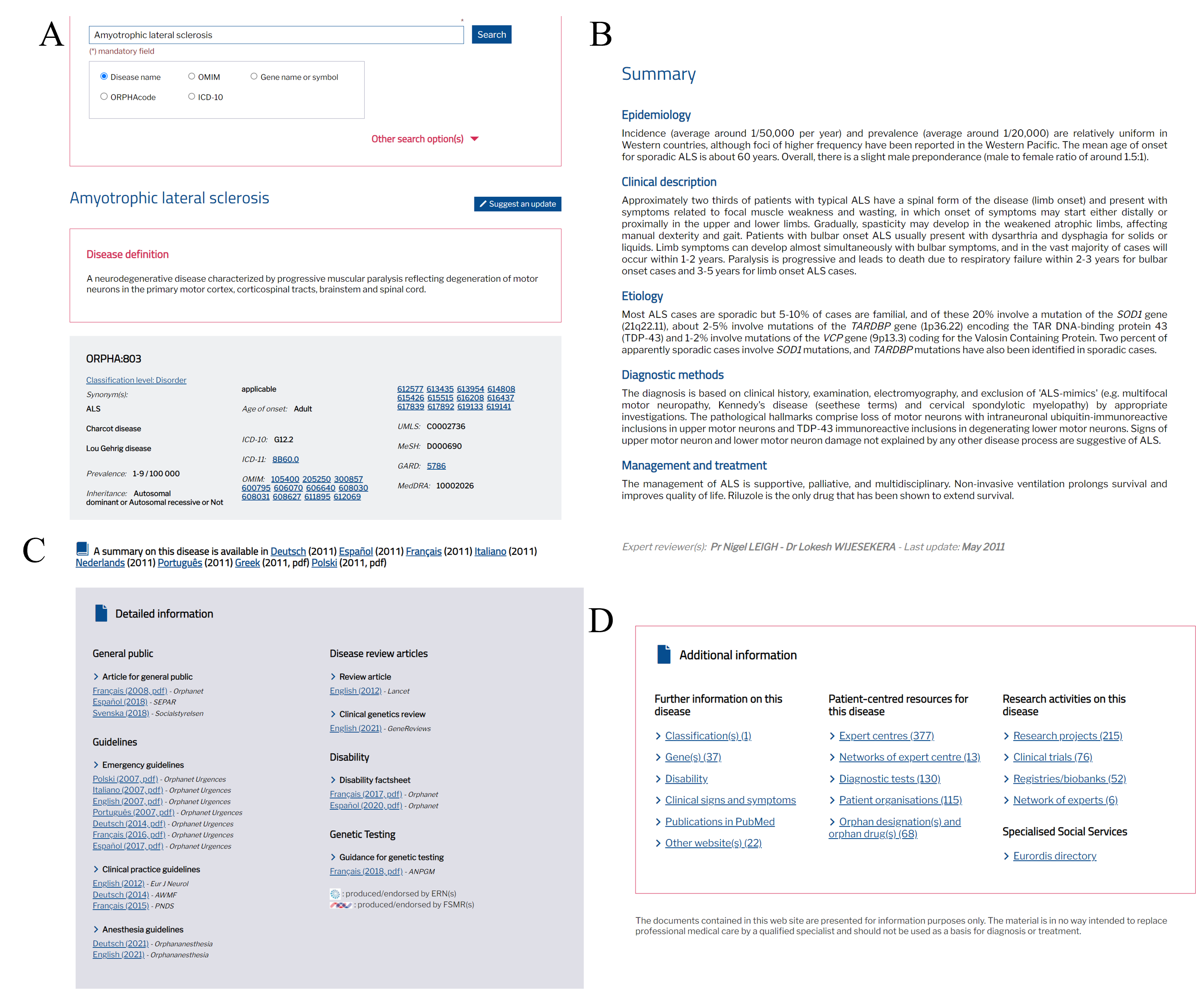


References:

Ando, R.K. BioCreative II gene mention tagging system at IBM Watson. In, *Proceedings of the Second BioCreative Challenge Evaluation Workshop*. Centro Nacional de Investigaciones Oncologicas (CNIO) Madrid, Spain; 2007. p. 101-103.

Bravo, À.*, et al.* Extraction of relations between genes and diseases from text and large-scale data analysis: implications for translational research. *BMC bioinformatics* 2015;16:1-17.

Gerner, M., Nenadic, G. and Bergman, C.M. LINNAEUS: a species name identification system for biomedical literature. *BMC bioinformatics* 2010;11(1):1-17.

Goodman, J.M.*, et al.* InChI version 1.06: now more than 99.99% reliable. *J Cheminform* 2021;13(1):40.

Gupta, H.*, et al.* RareDDB: An Integrated Catalog of Rare Disease Database. *Clin Med Biochemistry Open Access* 2016;2(111):2.

Hastings, J.*, et al.* ChEBI in 2016: Improved services and an expanding collection of metabolites. *Nucleic Acids Res* 2016;44(D1):D1214-1219.

Hoskins, A. Genetic and Rare Diseases Information Center (GARD). *Med Ref Serv Q* 2022;41(4):389-394.

Howe, K.L.*, et al.* Ensembl 2021. *Nucleic Acids Res* 2021;49(D1):D884-D891.

Jia, J.*, et al.* eRAM: encyclopedia of rare disease annotations for precision medicine. *Nucleic acids research* 2018;46(D1):D937-D943.

Kim, S.*, et al.* PubChem in 2021: new data content and improved web interfaces. *Nucleic Acids Res* 2021;49(D1):D1388-D1395.

Krallinger, M.*, et al.* The CHEMDNER corpus of chemicals and drugs and its annotation principles. *Journal of cheminformatics* 2015;7(1):1-17.

Kuo, T.C.*, et al.* RSDB: A rare skin disease database to link drugs with potential drug targets for rare skin diseases. *Sci Data* 2022;9(1):521.

Li, J.*, et al.* BioCreative V CDR task corpus: a resource for chemical disease relation extraction. *Database* 2016;2016.

Martens, M.*, et al.* WikiPathways: connecting communities. *Nucleic Acids Res* 2021;49(D1):D613-D621.

Putkowski, S. National Organization for Rare Disorders (NORD): providing advocacy for people with rare disorders. *NASN Sch Nurse* 2010;25(1):38-41.

Tweedie, S.*, et al.* Genenames.org: the HGNC and VGNC resources in 2021. *Nucleic Acids Res* 2021;49(D1):D939-D946.

Van Mulligen, E.M.*, et al.* The EU-ADR corpus: annotated drugs, diseases, targets, and their relationships. *Journal of biomedical informatics* 2012;45(5):879-884.

Weinreich, S.S.*, et al.* [Orphanet: a European database for rare diseases]. *Ned Tijdschr Geneeskd* 2008;152(9):518-519.

Wishart, D.S.*, et al.* DrugBank 5.0: a major update to the DrugBank database for 2018. *Nucleic Acids Res* 2018;46(D1):D1074-D1082.

Zhang, D.*, et al.* A Distant Supervision Corpus for Extracting Biomedical Relationships Between Chemicals, Diseases and Genes. *arXiv preprint arXiv:2204.06584* 2022.
